# Prospective multicentre cohort study of patient-reported outcomes and complications following major abdominal neoplastic surgery (PATRONUS) – study protocol for a CHIR-*Net* student-initiated German medical audit study (CHIR-*Net* SIGMA study)

**DOI:** 10.1186/s12893-018-0422-3

**Published:** 2018-10-29

**Authors:** Christoph A. Fink, Mirco Friedrich, Pia-Elena Frey, Lukas Rädeker, Alexander Leuck, Thomas Bruckner, Manuel Feisst, Solveig Tenckhoff, Christina Klose, Colette Dörr-Harim, Jens Neudecker, André L. Mihaljevic

**Affiliations:** 1 University of Heidelberg, Faculty of Medicine, Im Neuenheimer Feld 346, 69120 Heidelberg, Germany; 2 Institute of Medical Biometry and Informatics, University of Heidelberg, Im Neuenheimer Feld 130.3, 69120 Heidelberg, Germany; 3 CHIR-Net Coordination Centre at the Study Centre of the German Surgical Society (SDGC), University Hospital Heidelberg, Im Neuenheimer Feld 130.3, 69120 Heidelberg, Germany; 4 Department of Surgery, Campus Virchow-Klinikum, Charité, Universitätsmedizin Berlin, Augustenburger Platz 1, 13353 Berlin, Germany; 5 Department of General, Visceral and Transplantation Surgery, University Hospital Heidelberg, Im Neuenheimer Feld 110, 69120 Heidelberg, Germany

**Keywords:** Patient-reported outcome measures, Quality of life, Neoplasms, Postoperative complications, General surgery, Digestive system surgical procedures, CHIR-*Net*, SIGMA

## Abstract

**Background:**

One of the most important aspects of designing a clinical trial is selecting appropriate outcomes. Patient-reported outcomes (PROs) can provide a personal assessment of the burden and impact of a malignant disease and its treatment. PROs comprise a wide range of outcomes including basic clinical symptom scores and complex metrics such as health-related quality of life (HRQoL). There is limited data on how postoperative complications following cancer surgery affect symptoms and HRQoL. For this reason the primary aim of the PATRONUS study is to investigate how perioperative complications affect cancer-related symptoms and HRQoL in patients undergoing abdominal cancer surgery. The PATRONUS study is designed and will be initiated and conducted by medical students under the direct supervision of clinician scientists based on the concept of inquiry-based learning.

**Methods:**

PATRONUS is a non-interventional prospective multicentre cohort study. Patients undergoing elective oncological abdominal surgery will be recruited at regional centres of the clinical network of the German Surgical Society (CHIR-*Net*) and associated hospitals.

A core set of 12 cancer associated symptoms will be assessed via the PRO version of the Common Terminology Criteria for Adverse Events. The cancer-specific HRQoL will be measured via the computerised adaptive testing version of the European Organisation for Research and Treatment of Cancer (EORTC) QLQ-C30. PROs will be measured eight times over a period of six months. The short-term clinical outcome measure is the rate of postoperative complications (grade II to V) within 30 days according to the Clavien-Dindo classification. The long-term clinical outcome is overall survival within six months postoperative.

**Discussion:**

PATRONUS will provide essential insights into the patients’ assessment of their well-being and quality of life in direct relation to clinical outcome parameters following abdominal cancer surgery. Furthermore, PATRONUS will investigate the feasibility of multicentre student-led clinical research.

**Trial registration:**

German Clinical Trials Register: DRKS00013035 (registered on October 26, 2017).

Universal Trial Number (UTN): U1111–1202-8863.

**Electronic supplementary material:**

The online version of this article (10.1186/s12893-018-0422-3) contains supplementary material, which is available to authorized users.

## Background

Cancer is the second most common cause of death in Germany following cardiovascular diseases [1]. Due to demographic changes, the incidence and prevalence of malignancies in Germany will rise sharply within the next years [2–4]. According to German national data, the most frequent lethal tumour diseases include solid neoplasms of the abdominal cavity including pancreatic, liver, gastric and colorectal cancers [5]. For these tumours, surgery is the mainstay of curative as well as palliative treatment. At the same time, cancer patients will become older and comorbid [2, 6, 7]. Therefore patients, caregivers and specialists will face new challenges in the care of cancer patients. Accordingly the design and focus of clinical trials will need to adapt to this changing environment with an increasing need to integrate the patients’ perspective in clinical studies.

For this purpose, patient-reported outcomes (PROs) have been introduced next to survival and morbidity endpoints in oncological trials. PROs can be defined as ‘any outcome evaluated directly by the patient himself or herself and is based on the patient’s perception of a disease and its treatment(s)’ [8] and are measured via validated PRO measures (PROMs). PROMs in oncological trials can be classified into two main groups: PROMs for the assessment of cancer- and treatment-related symptoms and PROMs to measure health-related quality of life (HRQoL) [9]. Recently, two new PROMs have been proposed for each purpose: the EORTC has evaluated a digital, computer adaptive testing (CAT) version of their widely used cancer-specific HRQoL questionnaire C30 (CAT EORTC QLQ-C30) [10–12] and the National Cancer Institute (NCI) has validated a PROM version of the Common Terminology Criteria for Adverse Events (PRO-CTCAE) to measure cancer or treatment associated symptoms [13]. In addition, the NCI has defined a core-set of 12 symptoms that should be reported in all cancer trials [14].

PROs can provide additional benefit in clinical trials as they might provide a personal assessment of the burden of a malignant disease and its treatment and might complement survival, morbidity and safety data, thus enabling a full risk-benefit assessment [8]. Although PROs have been applied frequently in cancer trials, astonishingly little is known about the relationship between clinical endpoints and the well-being of cancer patients undergoing abdominal surgery. Arguably the most important clinical outcome in surgical oncology from a patient’s point of view is, apart from survival, the frequency and severity of complications. To this end the Clavien-Dindo classification which grades postoperative complications according to their sequelae has gained wide acceptance [15, 16]. Although it seems intuitive that complications adversely affect HRQoL and symptoms, the extent to which HRQoL and symptoms are affected is unknown. It is therefore also unclear whether complications considered more severe in the Clavien-Dindo classification system [15, 16], i.e. those with a higher severity grade, are related to reduced HRQoL or symptoms. Although several cross-sectional studies have evaluated HRQoL in patients who had undergone oncological abdominal surgery and found an association between postoperative complications and decreased long-term HRQoL [17–19], these results are questionable considering the design of these studies and their insufficient validity [20]. Most of the few available prospective trials have been performed exclusively in colorectal cancer surgery and show heterogeneous results, with some demonstrating no [21], some short-term [22] and some also long-term effects of complications on certain subdomains of HRQoL [23, 24]. For other cancers of the gastrointestinal tract, including pancreatic [25] and oesophageal cancer [26], data are even more sparse. Furthermore, the majority of these studies exhibit severe methodological limitations including single-centre design, limited number of patients, inadequate follow-up, and high rate of missing data. Finally, all of these studies used a wide range of different PROMs and frequently no standardised morbidity assessment. Therefore, a prospective multicentre study using validated complication measurements and PROMs seems urgently warranted.

### Student-led clinical research and learning-by-research

Although the principles of evidence-based medicine (EbM) are taught in most medical schools, the focus of most EbM curricula is on the application of EbM-principles in everyday clinical practice, but not on teaching scientific methodology or performing scientific work, i.e. generating new scientific evidence. Consequently, a future deficit in academic faculty seems likely in European countries [27–29].

Inquiry-based learning or learning-by-research are concepts that refer to a trend in higher education: to provide students with the opportunity to gain knowledge by conducting their own scientific inquiries or investigations. Although the underlying mechanisms still have to be revealed, projects to provide such research experiences are emerging and show that medical students can improve their clinical capabilities by contributing to student-initiated clinical trials [30–32]. Recently, a student-led initiative has successfully performed a number of large observational studies in a national cohort following gastrointestinal surgery demonstrating the feasibility of this concept [33, 34]. On an even larger scale the EuroSurg collaborative demonstrated the feasibility of this concept across 20 European countries [35]. However, all of these studies were confined to specific subpopulations of patients, excluded PROMs and investigated specific rather than all postoperative complications. The study idea for the PATRONUS study was conceived by the Study network of the German Surgical Society (CHIR-*Net*; www.chir-net.de). CHIR-*Net* has succeeded in creating a German-wide research infrastructure comprising university and non-university centres. Since its foundation in 2006, CHIR-*Net* has recruited more than 11,800 patients in randomised-controlled surgical trials and consists of 16 regional centres and their associated partner hospitals [36]. CHIR-*Net* has recently created a student-led clinical trial network (Student-initiated German Medical Audit, SIGMA; www.sigma.university) [37] which will run the PATRONUS study.

## Methods/design

This study protocol is written according to current SPIRIT guidelines [38]. The SPIRIT checklist is attached as Additional file 1.

### Study design

PATRONUS is a prospective multicentre single-arm observational cohort study across Germany.

### Outcome measures

#### Patient-reported outcome measures (PROMs)

- a core set of 12 cancer associated symptoms [14] (fatigue, insomnia, pain, anorexia, dyspnoea, cognitive problems, anxiety, nausea, depression, neuropathy, constipation, diarrhoea) measured via the PRO-CTCAE [13].
- cancer-specific HRQoL measured via the CAT EORTC QLQ-C30 [39, 40].

#### Clinical outcome measures

- Short-term clinical outcome measure is the rate of postoperative complications grade II (minor) and III-V (major) within 30 days according to the Clavien-Dindo classification [15].
- Long-term clinical outcome is overall survival within 6 months postoperative.

### Objectives

The primary aim of the study is to evaluate associations between the clinical outcomes and the two PROMs in the short-term (within 30 days) and long-term (after 3 and 6 months) in order to find out whether and to what extend complications (none/grade I vs. minor (grade II) vs. major (grade III-V)) influence the two PROMS in 5 predefined surgical cancer subgroups (upper-GI, pancreatic, hepatobiliary, colorectal, other abdominal cancers).

Further objectives are:

1. To describe the absolute values of a.) the newly defined CAT EORTC QLQ-C30 and b.) the set of 12 cancer-specific symptoms [14] measured via the PRO-CTCAE [13] for oncologic patients undergoing surgery at predefined times from baseline (preoperative) until 6 months postoperative in the 5 surgical cancer subgroups outlined above.
2. Although both of the proposed PROMs (PRO-CTCAE [13] and CAT version of the EORTC QLQ-C30) used in this trial have been validated, they are new and have never been tested head-to-head. Therefore, the existence of possible overlapping domains in both PROMs will be analysed.
3. In order to find out if a student-led multicentre observational study using an electronic data capture system is feasible.

For the primary objective and further objective 1. and 2., the two PROMs (PRO-CTCAE and CAT version of the EORTC QLQ-C30) in the short-term (within 30 days) and long-term (after 3 and 6 months), as well as is the rate of postoperative complications grade II (minor) and III-V (major) within 30 days according to the Clavien-Dindo classification and the overall survival within 6 months postoperative will be assessed.

For further objective 3 the following endpoints will be assessed:

- Rate of missing PRO data at 3 and 6 months
- Rate of missing clinical data at 30 days
- Number of participating trial sites including patients compared to initiated trial sites
- Rate of included patients in comparison to screened patients

Further endpoints can be found in the description of the study visits.

### Study duration and schedule

**Fig. 1:**
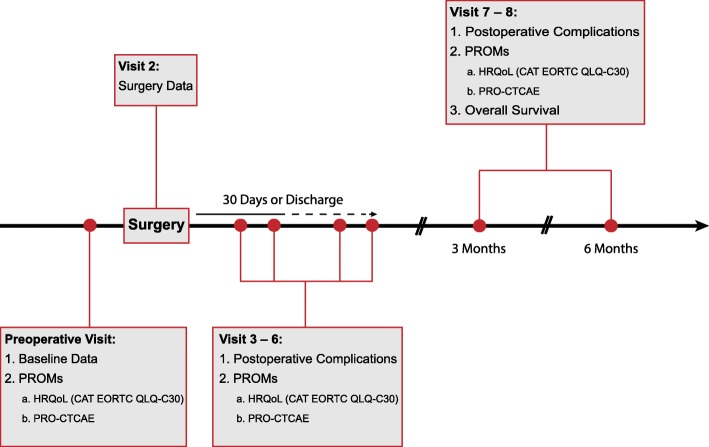
Patient flow chart. *CAT* computer adaptive testing version, *CTCAE* Common Terminology Criteria of Adverse Events, *EORTC* European Organisation for Research and Treatment of Cancer, *HRQoL* health-related quality of life, *PRO* patient-reported outcome, *PROM* patient reported outcome measure

The duration of the study for each patient is 6 months including follow-up. Milestones are listed in Table 1. The duration of the overall study is expected to be 18 months. Figure 1 displays the patient flow during the study.

**Table 1:** Milestones and study time plan

1	Preparation of trial, Ethic votes, database set-up	3 months
2	Initiation, training course for participants (including preparation) (overlap with 1)	1 month
3	Recruitment period	2 months
5	Follow-up period	6 months per patient
7	query management, database clearing	3 months
8	Report, publication	3 months
Total		18 months

### Study conduct and study centres

To enrol an adequate number of patients in the planned recruitment period, 30 hospitals including regional centres of the clinical trial network CHIR-*Net* (www.chir-net.de/regionalzentren-karte) as well as associated partner-hospitals will participate in the study.

A CHIR-*Net* SIGMA steering group for the PATRONUS study has been set up consisting of the authors of this publication and include the CHIR-*Net* coordination centre. This steering group has designed the study and is responsible for trial infrastructure, acquisition of centres, qualification of participating medical students and communication with centres and the public via digital tools (www.slack.com) and social media (www.facebook.com/sigmastudynetwork; https://twitter.com/sigmastudies).

At each trial site a so-called “mini-team” will be established consisting of an experienced clinical trialist (surgeon) and a group of at least two medical students. Furthermore, at each site a “local lead” will be appointed. The local lead is a medical student responsible for creating the mini-team, recruiting other students to the project, interacting with local surgeons and conducting the study. The local lead in conjunction with the associated surgeon is also responsible for gaining approval for the study from his/her local independent ethics committee according to the German Medical Association’s professional code (*Berufsordnung der Bundesärztekammer*).

All student participants will be trained prior to study initiation. To this end CHIR-*Net* SIGMA has developed an in-person and online clinical trials training programme (SIGMA clinical trial curriculum; SIGMA-CTC) which will be reported elsewhere.

### Interventions

No experimental or control intervention is tested as this is an observational, non-interventional prospective cohort study.

### Eligibility criteria

Every local team will recruit patients for the study independently. Inclusion criteria are: a.) patient’s age ≥ 18 years; b.) patient scheduled for elective abdominal surgery for confirmed or suspected malignancy; c.) patient’s ability to understand character of the study; d.) planned laparoscopic or open surgery or any variant (i.e. laparoscopic-assisted, laparoscopic-thoracoscopic), e.) written informed consent. Exclusion criteria are a.) language barrier that impedes follow-up or informed consent, b.) American Society of Anesthesiologists (ASA) grade ≥ 4.

All patients planned for an elective surgery for suspected or confirmed malignancy are asked for their participation in the trial if all inclusion criteria and none of the exclusion criteria are fulfilled. Screening lists are kept in order to analyse reasons for non-inclusion of patients and to calculate the case ascertainment rate.

### Patient timelines and description of study visits

Patients will be screened preoperatively (visit 1) by a member of the mini-team. Patients will be enrolled if they fulfil the inclusion/exclusion criteria and after having given their written informed consent after detailed patient information. Informed consent is sought by a member of the mini-team, i.e. medical student or clinical trialist (surgeon). Baseline data and the first set of PROMs are collected during screening/baseline visit. Surgical data is collected during visit 2. Short-term clinical outcome parameters and PROMs are collected during visits 3–6 within 30 postoperative days or until discharge. Long-term clinical data and PROMs are collected at visit 7 and 8. Detailed information about the collected data during the patients’ visits is displayed in Table 2 (SPIRIT figure).

**Table 2:** Adapted SPIRIT figure showing the visits and documented parameters of the PATRONUS study

Activity	Visit 1 (screening, consent)	Visit 2 (surgery)	Visit 3–6 (POD 3–5, 6–8, 10–14, 30–35 or at discharge)	Visit 6_TEL_ (POD 30–35 if patient has been discharged before)	Visit 7/8 (postoperative month 3 and 6)
expenditure of time per visit approx	20–25 min	–	15 min	15 min	15 min
Inclusion/exclusion criteria	X				
Informed consent	X				
Demographics and baseline data	X				
Documentation of PROMs: a.) 12 symptoms PRO-CTCAE* b.) cancer-specific HRQoL**	X		X^§^	X	X
Surgery		X			
Postoperative morbidity***			X	X	
SSI^$^			X	X	
Length of hospital stay			X	X	X
Reoperations			X	X	X
(Re)admissions			X	X	X
Overall survival			X	X	X

* 12 symptoms as recommended by the National Cancer Institute: fatigue, insomnia, pain, anorexia, dyspnoea, cognitive problems, anxiety, nausea, depression, neuropathy, constipation, diarrhoea assessed via PRO-CTCAE™

** according to the EORTC quality of life questionnaire C30

*** according to Clavien-Dindo

^$^ Documentation of SSI according to CDC [40, 41]

^§^ HRQoL once only on POD 30–35 or at discharge (Visit 6)

TEL Visit can be performed by telephone

*SSI* surgical site infection, *CDC* Centers of Disease Control and Prevention, *POD* postoperative day, *OS* overall survival, *PROM* patient-reported outcome measure, *CTCAE* Common Terminology Criteria of Adverse Events, *HRQoL* health-related quality of life

#### Visit 1 (preoperative, informed consent)

After informed consent, the following data items will be collected during visit 1: a.) demographic data; c.) baseline data d.) previous abdominal surgeries; e.) medical history; f.) HRQoL according to CAT EORTC QLQ-C30 [39, 40]; g.) PRO-CTCAE [13] of 12 core cancer-associated symptoms [14].

#### Visit 2 (surgery)

During surgery, the following parameters will be recorded: a.) duration of surgery; b.) estimated blood loss; c.) intraoperative blood transfusion; d.) surgery performed including completeness of macroscopic tumour resection; e.) category of surgery (upper-GI, pancreatic, hepatobiliary, colorectal, other); f.) degree of contamination according to the Centers for Disease Control and Prevention (CDC) definition [41].

#### Visit 3 (postoperative day 3–5)

This visit is performed to document short-term clinical outcome (complication rate). The following data items need to be documented: a.) survival; b.) discharge; c.) PRO-CTCAE symptoms; d.) reoperation(s); e.) complications according to Clavien-Dindo; f.) surgical site infections (SSI) including grading according to the CDC classification [41]; g.) weight.

#### Visit 4 (postoperative day 6–8)

Visit 4 has an equivalent content to visit 3, with the exception of the PRO-CTCAE symptoms, which are NOT documented during visit 4. If the patient is discharged before day 6–8, visit 4 is omitted.

#### Visit 5 (postoperative day 10–14)

Visit 5 has an equivalent content to visit 3. If the patient is discharged before day 10, visit 5 is omitted.

#### Visit 6 (postoperative day 30–35 or at discharge)

Visit 6 is performed once between postoperative day 30–35 or at discharge if the patient leaves before day 30. The following data items need to be documented: a.) survival; b.) PRO-CTCAE symptoms; c.) CAT EORTC QLQ-C30; d.) reoperation(s); e.) complication assessment according to the Clavien-Dindo classification; f.) SSI assessment according to the CDC classification; g.) weight.

#### Visit 6_TEL_ (postoperative day 30–35)

Visit 6_TEL_ is performed if the patient has been discharged before postoperative day 30–35. If the patient is still in hospital on day 30–35 visit 6_TEL_ is omitted. The visit should be conducted between day 30–35. This visit can be performed in person (outpatient clinic) or, if the patient cannot be seen in person, on the phone. The following data items have to be collected: a.) survival; b.) PRO-CTCAE symptoms; c.) CAT EORTC QLQ-C30; d.) reoperation(s); e.) (re-)hospitalization; f.) complication assessment according to the Clavien-Dindo classification; g.) SSI assessment according to the CDC classification; h.) details of postoperative oncological treatment; i.) weight.

#### Visit 7/8 (postoperative month 3/6)

Visit 7 and 8 have equivalent contents. Visit 7 and 8 can be performed in person (in person follow-up) or as a telecommunication visit (e.g. phone). The following data items need to be recorded: a.) survival; b.) PRO-CTCAE; c.) CAT EORTC QLQ-C30; d.) weight; e.) reoperation(s); f.) (re-)hospitalization; g.) oncologic treatment; h.) complication(s) according to the Clavien-Dindo classification.

#### Event Visit_1-x_ (anytime between visit 1–8)

Event visits are optional, i.e. they do not have to be performed but can be performed if necessary. The objective is to document survival, complications and/or PROs anytime during the study, i.e. between visit 1–8, if they are not covered by the other visits. The following data items should be collected: a.) date; b.) PRO-CTCAE; c.) CAT EORTC QLQ-C30; d.) survival; e.) reoperation(s); f.) hospitalization/readmission; g.) SSI assessment according to the CDC classification; h.) complication assessment according to the Clavien-Dindo classification; i.) oncological treatment; j.) weight.

### Risk-benefit assessment

As this is a non-interventional, observational study, no beneficial treatment effect is to be expected. Accordingly, no additional risk is expected for the individual participant. However, patients might benefit from more frequent and more thorough postoperative clinical visits by the local mini-teams. Furthermore, the study allows the patients to measure and feedback a number of PROMs which is not available for non-participating patients. During digital PROM evaluation patients have the opportunity to contact their local study team. Finally, the results of the study could benefit future patients by integrating and improving PROMs in the clinical treatment of cancer patients. Given the risk-benefit assessment no ancillary or post-trial care or compensation to those who suffer harm from the study is planned.

### Ethical and legal aspects, informed consent

The PATRONUS study will be conducted in agreement with the Declaration of Helsinki in its current version. This study protocol has been written and the study will be conducted and analysed in accordance with relevant national and international rules and regulations including international conference of harmonization good clinical practice (ICH-GCP) guidelines.

All patients will be informed of the aims and conduct of the study to allow an individual risk-benefit assessment based on informed consent. It will be emphasised that the participation is voluntary and that the patient is allowed to refuse further participation in the study whenever he/she wants to. In case of withdrawal from the study the patient will be asked whether her/his previously collected data may be used for analysis or not. In the latter case, all pseudonymised data entered in the eCRF will be deleted.

It is the responsibility of the investigator/medical student to maintain patients’ confidentiality. During the study, patients will be identified solely by means of their individual identification code. Study specific documents and patient data will be stored in accordance with local data protection law/ ICH-GCP Guidelines at the local trial sites and will be handled in strict confidence and in line with the obligations of medical secrecy, the EU General Data Protection Regulation (*Datenschutzgrundverordnung*), the Federal Data Protection Act (*Bundesdatenschutzgesetz*) and the State Data Protection Act (*Landesdatenschutzgesetz*). Participating patients’ data will be recorded in pseudonymised form.

The trial may be prematurely closed by the coordinating investigators in consultation with the responsible statistician and the steering committee. Reasons that may necessitate termination of the study include a.) it appears that patients’ enrolment is unsatisfactory with respect to quality and/or quantity or data recording is severely inaccurate and/or incomplete; b.) External evidence demanding a termination of the trial. The independent ethics committee (IEC) must then be informed.

The results of the trial will be published in a peer-reviewed journal under the authorship of CHIR-*Net* SIGMA. All participating personel will be listed with their specific contribution. Results will be distributed to the public via www.sigma.university, www.facebook.com/sigmastudynetwork and https://twitter.com/sigmastudies.

### Data management

An electronic case report form (eCRF) will be used for data collection. To assure a safe and secure environment for data acquired, the RedCap™ system (www.project-redcap.org) will be used for remote data entry [42]. RedCap™ is validated and compliant with FDA 21 CRF part 11. Data transmission is encrypted with secure socket layer (SSL) technology. The database server is located in a secure data centre at the University of Heidelberg, Germany and is protected by a firewall. The system provides an infrastructure to support user roles and rights. Only authorised users are able to enter or edit data, the access is restricted to data of the patients in the respective centre. All changes to data are logged with a computerized timestamp in an audit trail. All clinical data will be pseudonymized. Backups are conducted regularly.

All protocol-required information collected during the study will be entered by an authorised member of the mini-team in the eCRF. For HRQoL and PRO-CTCAE results, patients may directly enter the data in the eCRF. Any outstanding entries must be completed immediately after the final examination. An explanation should be given for all missing data. The completed eCRF must be reviewed and signed by an authorised member of the mini-team.

In order to guarantee quality, validity and plausibility of data, a data validation plan is implemented which uses real-time edit-checks and validating programs, which will generate queries. The investigator, medical student or the designated representatives are obliged to clarify the edit-checks and queries.

All data will be integrated in a statistical analysis system. The data access is restricted to the data manager and the biometricians responsible for the trial. The data will be managed and analysed in accordance with the appropriate Standard Operating Procedures valid in the Institute of Medical Biometry and Informatics at the University of Heidelberg.

A study-specific data validation plan has been established ensuring that implausibility of data, missing data and imbalances between centers, e.g. reporting of postoperative complications are revealed. In this case, queries will be sent to the centres and need to be resolved by the mini-teams. In the PATRONUS study only a limited on-site monitoring will be performed. An external monitor will check, whether signed informed-consent forms are available for all patients.

### Statistical procedures

Since this is an exploratory study, all analyses will be descriptive and *p*-values < 0.05 will be referred to as statistically significant in descriptive sense. The patient characteristics and outcomes will be described for the whole cohort and for the different subgroups divided according to tumour entity with appropriate descriptive statistics (mean, standard deviation, median, interquartile range, minimum, maximum in case of continuous data and scores, or absolute and relative frequencies in case of categorical data).

To analyse the association between perioperative complications (Clavien-Dindo grading) and the set of 12 PRO-CTCAE symptoms as well as the subscales of CAT EORTC QLQ-C30 in the short- and long-term, Spearman’s rank-correlation coefficients (Spearman’s rho) will be calculated and analysis of variance will be performed.

For the description of the absolute values of the CAT EORTC QLQ-C30 as well as the set of 12 cancer-specific symptoms measured via the PRO-CTCAE appropriate descriptive measures will be reported.

Possible overlapping between the two PROMs (PRO-CTCAE and CAT EORTC QLQ-C30) will be analysed calculating Spearman’s rhos between the respective scales.

The rates of missing PRO data, clinical data and the rate of included patients compared to screened patients will be calculated and reported with adequate descriptive measures. The number of participating trial sites including patients compared to initiated trial sites and the rate of included patients in comparison to screened patients per trial site will be calculated, as well. The achievement of the respective goal rates will be evaluated by binomial tests.

Further analysis includes correlations between ASA score, comorbidities, neoadjuvant therapy, socio-economic factors, smoking, alcohol consumption and other baseline factors and subscales of the two PROMs and complications. Survival analysis includes Kaplan-Meier graphs and log-rank-tests between different tumour entities. Cox regression analysis will be performed to evaluate possible relationships between survival and different baseline covariates.

Wherever appropriate, graphics will be created to illustrate the data and findings. Details of the statistical analysis will be described in the statistical analysis plan, which will be written prior to the database closure. For the statistical analysis, IBM SPSS Statistics Version 24 (or higher) and SAS (SAS Institute, Cary, NC, USA) version 9.4 (or higher) will be used. No interim analysis is planned.

### Sample size calculation

Since this is an exploratory cohort study, no formal sample size calculation was done. Assuming 30 clinics to participate and a recruiting rate of about evaluable 30 patients per centre, we assume to have the data of about 900 patients to evaluate. This sample size is big enough to give answers with a good precision overall and in specific subgroups such as different cancer entities.

### Missing values

Due to the nature of the study (observational cohort study) no imputation of missing values will be provided. Appearance and frequency of missing data is examined in line with the objectives of the study.

## Discussion

Given the potential advantages of PROMs in clinical trials [8, 43] astonishingly little focus has been put on PROMs in surgical oncology. Here PROMs are frequently analysed inadequately (e.g. not addressing missing data) and are rarely the main focus of investigation. Consequently, very little is known about the relationship of clinical endpoints including postoperative surgical complications and PROMs in surgical oncology. In addition, previous studies investigating the relationship between PROMs and complications in abdominal cancer surgery have frequently used a cross-sectional design with inadequate validity [17–19] and results are thus questionable [20]. Moreover, most studies have focused on colorectal cancer surgery only, have reported conflicting results and lacked standardized complication measurements.

Similar to our planned PATRONUS study Bosma et al. have used a prospective observational design to analyse patients undergoing colorectal surgery and found an association between the severity of complications (measured via Clavien-Dindo) and HRQoL within the first 6 weeks, but not after 12 months [22]. However, this study had a single-centre design, used the short version of the WHO HRQoL assessment instrument [44], which covers no cancer-specific symptoms and is a generic, i.e. not cancer-specific HRQoL tool. In contrast, Di Cristofaro et al. reported no significant relationship between complications and global HRQoL (EORTC QLQ-C30, -CR29) in their prospective trial of 166 patients in 3 centres undergoing colorectal cancer surgery [21]. The randomised-controlled CLASSIC trial (laparoscopic vs. open resection for colorectal cancer) reported yet another result by prospectively analysing the HRQoL (generic EQ5D and cancer-specific EORTC QLQ-C30/CR38) in 614 randomised patients [23]. In contrast to the previous trials, significant long-term differences in QoL between patients with and without complications were found for Physical and Social Function, Role Functioning, and Body Image on EORTC QLQ-C30/QLQ-CR38 analysis and Mobility, Self-care, and Pain/Discomfort on EQ5D analysis. In line with these findings Anthony et al. reported an association with postoperative complications (no standardised evaluation) and postoperative HRQoL at 12 months using the Functional Assessment of Cancer Therapy (FACT-C) tool in 63 patients undergoing open colorectal cancer surgery [24]. For other types of abdominal cancer entities, results are even rarer including the study by Heerkens et al. who found no statistically significant and clinically relevant differences in HRQoL (using the EORTC QLQ-C30, -PAN26 and RAND26 questionnaire) between patients with severe compared to no/mild complications following pancreatic surgery in their single centre study [25] and Rutegard et al. who has analysed the Swedish national cancer registry for patients undergoing oesophagectomy for cancer and reported significantly impaired HRQoL (EORTC QLQ-C30 and -OES18) at 6 months in patients who had experienced technical surgical complications [26].

In summary, studies investigating the relationship between postoperative complications and PROMs are sparse and many studies exhibit severe methodological shortcomings. In addition, studies have used a multitude of different PROMs impeding direct comparison of results. In the meantime, clear recommendations exist for oncological trials to focus on two PROMs, namely HRQoL (either generic, cancer-specific or cancer-type specific) and cancer specific symptoms [8, 14]. For the latter the NCI has recently published recommendations which cancer-specific symptoms should be evaluated in all cancer patients (core set of 12 symptoms) [14], but has not specified the tools that should be applied to measure these symptoms. The EORTC QLQ-C30 has become one of the most widely used tools to measure cancer-specific HRQoL over the last decades [39] and recently a computerised adaptive version has been developed but not yet tested in surgical oncology. Similarly, the PRO-CTCAE symptoms score might be a fitting candidate to assess the proposed core set of cancer symptoms but again its evaluation in surgical oncology is pending. Therefore, a prospective multicentre study using an accepted complication measurement (Clavien-Dindo classification) and the two new PROMs as planned in our PATRONUS study seems urgently warranted.

Furthermore, a direct comparison of the two tools in overlapping domains (cancer-symptoms) is pending given their so far limited application in oncology. Again this issue will be studied in our study.

Although PROMs have numerous potential advantages their true benefit has been limited and PROMs have ‘[…] rarely been informative from a licensure perspective’ [8]. The reasons for this are methodological aspects of PROMs that are frequently not adequately addressed in clinical trials including a.) inadequate PROM development [43]; b.) inadequate psychometric reliability, validity and responsiveness [43, 45]; c.) lack of an a priori specification of the expected effect size, PRO domain under investigation and sample size calculation [8] and d.) missing data, adding to attrition bias and necessitating the a priori specification of imputation methods [46]. While the first two aspects are not a concern for the two PROMs selected for our study, sample size calculation and missing data might be a concern, especially in surgical oncology with patients experiencing complications being less likely able to fill out PROMs. PATRONUS will create a sound database for sample size calculation for future clinical trials and will also offer a good estimate of the rate of missing data which can be expected in the context of surgical oncology and digital PROM assessment.

An important aspect of the PATRONUS study is the design and conduct by medical students. PATRONUS allows medical students to interact with concepts and methods of clinical research thereby potentially inspiring their orientation towards academic careers and work as clinician scientists [27, 28]. Although the conduct of multicentre clinical observational studies is relatively new, previous examples in the UK have shown the feasibility of this concept [33]. However, PATRONUS will add some important aspects compared to previous studies as patients will need to undergo written informed consent, PRO evaluation is performed, the follow-up is significantly longer and the study design more complex.

The limitations of the PATRONUS study are numerous. First, it is a single-arm observational study and therefore cannot elucidate a causal relationship. It will, however, serve to generate hypotheses for future clinical research.

Second, data quality might be a concern. It is unclear if the assessment of postoperative complications performed by medical students is comparable to the assessment of trained doctors or study nurses. However, the SIGMA study group has implemented a number of measures to ensure high quality data assessment including a data validation plan (see methods section), centralized monitoring and training of participating study personnel in the SIGMA clinical trials curriculum prior to study initiation. Briefly, this curriculum includes a.) an obligatory e-learning session on the Clavien-Dindo classification; b.) lectures on the basics of evidence based medicine, levels of evidence, study types, statistical analysis plan, ethics, laws and regulatory obligations, Good Clinical Practice, and basics of data management; c.) workshops on acquiring informed consent from patients with actors and video-feedback, on how to improve patient recruitment, RedCap database training and bedside-teaching with Clavien-Dindo assessment. Within the PATRONUS study, only limited onsite monitoring will be done. An external monitor will check whether signed informed consent forms are available from all patients. However, no source data verification will be implemented. This is in contrast to the more enhanced data verification plans of other related study projects (e.g. EuroSurg Imagine trial) [47].

Third, as pointed out above the rate of SSIs according to CDC will be assessed as a secondary outcome parameter in our study. It is known from the analysis of RCTs, that there is an underreporting of SSIs if assessed as secondary endpoints compared to the assessment as primary endpoint [48]. Nonetheless, as SSI are among the most frequent postoperative complications assessment seems necessary both from an education and clinical perspective.

Fourth, as this is the first student-led study in Germany and of the CHIR-*Net* SIGMA group, feasibility and recruitment is unclear. Finally, many aspects of the concept of student-led clinical research warrant further evaluation.

### Trial status

Ethical approval was granted by the independent ethics committee of the Medical Faculty of the University of Heidelberg on 11^th^ September 2017 (version 1.1; reference S-466/2017) and patient recruitment started in February 2018.

## Additional file

Additional file 1: SPIRIT 2013 Checklist: Recommended items to address in a clinical trial protocol and related documents. (DOCX 62 kb)

## Abbreviations

(e)CRF: (electronic) Case Report Form
ASA: American Society of Anesthesiologists
CAT: Computer adaptive testing
CDC: Centers for Disease Control and Prevention
CHIR-*Net*: Clinical trial network of the German Surgical Society
CTCAE: Common Terminology Criteria of Adverse Events
DRKS: Deutsches Register Klinische Studien (German Clinical Trials Register)
EbM: Evidence-based Medicine
EORTC: European Organisation for Research and Treatment of Cancer
HRQoL: Health-related quality of life
NCI: U.S. National Cancer Institute
POD: Post-Operative Day
PRO: Patient Reported Outcome
PROM: Patient Reported Outcome Measure
QLQ: Quality of Life Questionnaire
SAE: Serious Adverse Event
SIGMA: Student-Initiated German Medical Audit
SIGMA-CTC: SIGMA Clinical Trial Curriculum
SSI: Surgical Site Infection
